# Supplementary material for: The Risk of Pyelonephritis Following Uncomplicated Cystitis: A Nationwide Primary Healthcare Study
Source: Antibiotics (Basel). 2022 Nov 24;11(12):1695. doi: 10.3390/antibiotics11121695 (PMC9774091; doi:10.3390/antibiotics11121695)

Supplementary

The Risk of Pyelonephritis following Uncomplicated Cystitis: A Nationwide Primary Healthcare Study

Filip Jansåker [1,2,*], Xinjun Li [1], Ingvild Vik [3,4], Niels Frimodt-Møller [2], Jenny Dahl Knudsen [2] and Kristina Sundquist [1,5,6]

Table S1. The study population of women with acute uncomplicated cystitis and acute pyelonephritis (outcome) diagnosed in outpatient specialist care or in primary healthcare settings within 30 days and within 90 days from the cystitis event (2006-2018)

Table S2. The association between antibiotic treatment (redeemed within five days) for acute uncomplicated cystitis and acute outpatient pyelonephritis diagnosed within 30 days from the cystitis event, adjusted for sociodemographic factors, cervical cancer, and parity (2006-2018)

Table S3. The association between antibiotic treatment (redeemed within five days) for acute uncomplicated cystitis and acute outpatient pyelonephritis diagnosed within 90 days from the cystitis event, adjusted for sociodemographic factors, cervical cancer, and parity (2006-2018)

Table S4. Descriptive data and sensitivity analysis on the association between antibiotic treatment (redeemed within two days) for acute uncomplicated cystitis and acute outpatient pyelonephritis within 7 days and 30 days from the cystitis event (2006-2018)

Table S5. Sensitivity analysis on the association between antibiotic treatment (redeemed within two days) for acute uncomplicated cystitis and acute outpatient pyelonephritis within 7 days and 30 days from the cystitis event, adjusted for sociodemographic factors, cervical cancer, and parity (2006-2018)

Table S6. The study population of women with acute uncomplicated cystitis and hospitalization due to acute pyelonephritis (outcome) diagnosed within 30 days and 90 days from the cystitis event (2006-2018)

Table S7. The association between antibiotic treatment (redeemed within five days) for acute uncomplicated cystitis and hospitalization due to acute pyelonephritis within 30 days from the cystitis event, adjusted for sociodemographic factors, cervical cancer, and parity (2006-2018)

Table S8. The association between antibiotic treatment (redeemed within five days) for acute uncomplicated cystitis and hospitalization due to acute pyelonephritis within 90 days from the cystitis event, adjusted for sociodemographic factors, cervical cancer, and parity (2006-2018)

Figure S1. Flowchart of the eligibility criteria and sampling of the study population of women with a diagnose (first occurrence) of acute uncomplicated cystitis in nationwide primary healthcare data (1st January 2006 to 2nd October 2018) and follow-up for pyelonephritis (31st December 2018)

Figure S2. The 30-day risk of acute outpatient pyelonephritis after acute uncomplicated cystitis treated with antibiotics or not (redeemed within two days)
(survival probability 1.00 = no cases of pyelonephritis)

Figure S3. The 30-day risk of acute outpatient pyelonephritis after acute uncomplicated cystitis treated with antibiotics or not (redeemed within five days)
(survival probability 1.00 = no cases of pyelonephritis)

Figure S4. The 30-day risk of hospitalization due to acute pyelonephritis after acute uncomplicated cystitis treated with antibiotics or not (redeemed within five days)
(survival probability 1.00 = no cases of pyelonephritis)

Table S1. The study population of women with acute uncomplicated cystitis and acute pyelonephritis (outcome) diagnosed in outpatient specialist care or in primary healthcare settings within 30 days and within 90 days from the cystitis event (2006-2018)

	Total population		With acute pyelonephritis within:					
			30 days			90 days		
	No.	Proportion %	No.	Proportion %	Case rate %	No.	Proportion %	Case rate %
Including the 3585 women with pyelonephritis prior to antibiotic treatment in the no antibiotic treatment group								
**No antibiotic treatment**	408 281	54.3	5829	78.2	1.43	6574	77.6	1.61
**Any antibiotic**[a]	344 008	45.7	1625	21.8	0.47	1898	22.4	0.55
**All**	752 289	100.0	7454	100.0	0.99	8472	100.0	1.13
Excluding the 3585 women with pyelonephritis prior to antibiotic treatment								
**No antibiotic treatment** (in analysis)	404 696	54.1	2244	58.0	0.55	2989	61.2	0.74
**Any antibiotic**[a]	344 008	45.9	1625	42.0	0.47	1898	38.8	0.55
Penicillins with extended spectrum (J01CA)	201 114	26.9	490	12.7	0.24	643	13.2	0.32
Nitrofuran derivatives (J01XE)	85 238	11.4	177	4.6	0.21	235	4.8	0.28
Trimethoprim and derivatives (J01EA)	35 361	4.7	51	1.3	0.14	63	1.3	0.18
Fluoroquinolones (J01MA)	17 079	2.3	828	21.4	4.85	868	17.8	5.08
Cephalosporins (J01DB-E,I)	4136	0.6	37	1.0	0.89	44	0.9	1.06
Others[b]	1080	0.1	42	1.1	3.89	45	0.9	4.17
**Age groups (years)**								
18-24	156 506	20.9	858	22.2	0.55	1135	23.2	0.73
25-34	157 301	21.0	776	20.1	0.49	986	20.2	0.63
34-44	140 796	18.8	721	18.6	0.51	857	17.5	0.61
45-65	294 101	39.3	1514	39.1	0.51	1909	39.1	0.65
**Educational level**								
<12	465 996	62.2	2458	63.5	0.53	3118	63.8	0.67
> 12	282 708	37.8	1411	36.5	0.50	1769	36.2	0.63
**Family income**								
Low	179 204	23.9	848	21.9	0.47	1076	22.0	0.60
Middle	379 633	50.7	1998	51.6	0.53	2510	51.4	0.66
High	189 867	25.4	1023	26.4	0.54	1301	26.6	0.69
**Region of residence**								
Large cities	481 650	64.3	2451	63.3	0.51	3089	63.2	0.64
Southern Sweden	194 194	25.9	975	25.2	0.50	1259	25.8	0.65
Northern Sweden	72 860	9.7	443	11.4	0.61	539	11.0	0.74
**Country of origin**								
Sweden (born in)	595 586	79.5	3142	81.2	0.53	3965	81.1	0.67
Eastern Europe	36 259	4.8	186	4.8	0.51	229	4.7	0.63
Western countries	31 363	4.2	153	4.0	0.49	188	3.8	0.60
Middle East/North Africa	42 756	5.7	166	4.3	0.39	234	4.8	0.55
Africa (excluding North Africa)	11 845	1.6	53	1.4	0.45	61	1.2	0.51
Asia (excl. Middle East) and Oceania	20 320	2.7	110	2.8	0.54	138	2.8	0.68
Latin America and the Caribbean	10 575	1.4	59	1.5	0.56	72	1.5	0.68
**Parity**								
No child	318 340	42.5	1674	43.3	0.53	2153	44.1	0.68
With child	430 364	57.5	2195	56.7	0.51	2734	55.9	0.64
**Cervical cancer**								
Non	727 711	97.2	3743	96.7	0.51	4731	96.8	0.65
Yes	20 993	2.8	126	3.3	0.60	156	3.2	0.74
**All**	**748 704**	**100.0**	**3869**	**100.0**	**0.52**	**4887**	**100.0**	**0.65**

a: within five days from the cystitis event and before the pyelonephritis event. * 3585 women were excluded due to diagnosis of outcomes before antibiotic treatment. b: Others included ATC-codes J01MB, J01EB, J01EC, J01ED, J01EE, and J01CR. Nationwide primary healthcare data was used to identify the study population of women with uncomplicated cystitis. The Swedish Prescribed Drug Register was used to identify antibiotic treatment. Nationwide primary healthcare data and the National Patient Register (Out-patient data) were used to identify pyelonephritis.

Table S2. The association between antibiotic treatment (redeemed within five days) for acute uncomplicated cystitis and acute outpatient pyelonephritis diagnosed within 30 days from the cystitis event, adjusted for sociodemographic factors, cervical cancer, and parity (2006-2018)

	Model 1				Model 2				Model 3			
Covariates	OR	95% CI		P-value	OR	95% CI		P-value	OR	95% CI		P-value
**Antibiotic (ref. Non)**	0.85	0.80	0.91	<.0001	0.85	0.80	0.91	<.0001	0.85	0.80	0.91	<.0001
**Age (ref. age 18-24 years)**												
25-34					0.91	0.82	1.00	0.0585	0.91	0.82	1.02	0.0930
34-44					0.94	0.85	1.04	0.1985	0.95	0.85	1.07	0.3927
45-65					0.91	0.83	0.99	0.0298	0.93	0.84	1.03	0.1393
**Educational level (ref. > 12 years)**					1.06	0.99	1.13	0.1176	1.06	0.99	1.13	0.1172
**Family income (ref. High)**												
Low					0.88	0.80	0.97	0.0110	0.88	0.80	0.98	0.0136
Middle					0.96	0.89	1.04	0.2813	0.96	0.89	1.04	0.2997
**Region of residence (ref. Large cities)**												
Southern Sweden					0.97	0.90	1.05	0.4129	0.97	0.90	1.05	0.4207
Northern Sweden					1.17	1.05	1.29	0.0034	1.17	1.05	1.29	0.0034
**Country of origin (ref. Born in Sweden)**												
Eastern Europe					1.02	0.88	1.19	0.7939	1.02	0.88	1.18	0.8233
Western countries					0.95	0.81	1.12	0.5763	0.95	0.81	1.12	0.5648
Middle East/North Africa					0.78	0.67	0.92	0.0030	0.78	0.67	0.92	0.0031
Africa (excluding North Africa)					0.89	0.68	1.17	0.4044	0.89	0.68	1.17	0.4045
Asia (excluding Middle East) and Oceania					1.08	0.89	1.31	0.4463	1.08	0.89	1.31	0.4532
Latin America and the Caribbean					1.10	0.85	1.42	0.4823	1.10	0.85	1.42	0.4905
**Cervical cancer (ref. Non)**									0.98	0.91	1.06	0.5686
**Parity (ref. Non)**									1.14	0.95	1.37	0.1479

CI: Confidence interval. OR: Odds ratio. Nationwide primary healthcare data was used to identify the study population of women with uncomplicated cystitis. The Swedish Prescribed Drug Register was used to identify antibiotic treatment. Nationwide primary healthcare data and the National Patient Register (Out-patient data) were used to identify pyelonephritis. Model 1: Univariate. Model 2: Adjusted for sociodemographic factors. Model 3: Fully adjusted for all covariates.

Table S3. The association between antibiotic treatment (redeemed within five days) for acute uncomplicated cystitis and acute outpatient pyelonephritis diagnosed within 90 days from the cystitis event, adjusted for sociodemographic factors, cervical cancer, and parity (2006-2018)

	Model 1				Model 2				Model 3			
Covariates	OR	95% CI		P-value	OR	95% CI		P-value	OR	95% CI		P-value
**Antibiotic (ref. Non)**	0.75	0.70	0.79	<.0001	0.75	0.70	0.79	<.0001	0.75	0.70	0.79	<.0001
**Age (ref. age 18-24 years)**												
25-34					0.86	0.79	0.94	0.0008	0.87	0.79	0.95	0.0027
34-44					0.83	0.76	0.91	<.0001	0.85	0.76	0.94	0.0014
45-65					0.85	0.79	0.92	<.0001	0.87	0.80	0.95	0.0028
**Educational level (ref. > 12 years)**					1.06	1.00	1.12	0.0724	1.06	1.00	1.12	0.0708
**Family income (ref. High)**												
Low					0.86	0.79	0.94	0.0011	0.87	0.79	0.95	0.0015
Middle					0.94	0.88	1.01	0.0848	0.94	0.88	1.01	0.0943
**Region of residence (ref. Large cities)**												
Southern Sweden					0.98	0.92	1.05	0.6194	0.98	0.92	1.05	0.6312
Northern Sweden					1.11	1.01	1.22	0.0242	1.11	1.01	1.22	0.0243
**Country of origin (ref. Born in Sweden)**												
Eastern Europe					1.01	0.88	1.15	0.9103	1.00	0.88	1.15	0.9522
Western countries					0.94	0.81	1.09	0.4400	0.94	0.81	1.09	0.4242
Middle East/North Africa					0.89	0.77	1.02	0.0813	0.88	0.77	1.01	0.0788
Africa (excluding North Africa)					0.82	0.64	1.06	0.1358	0.82	0.64	1.06	0.1333
Asia (excluding Middle East) and Oceania					1.10	0.92	1.30	0.2877	1.10	0.92	1.30	0.2968
Latin America and the Caribbean					1.08	0.85	1.36	0.5398	1.07	0.85	1.36	0.5531
**Cervical cancer (ref. Non)**									0.97	0.91	1.04	0.4532
**Parity (ref. Non)**									1.11	0.95	1.31	0.1978

CI: Confidence interval. OR: Odds ratio. Nationwide primary healthcare data was used to identify the study population of women with uncomplicated cystitis. The Swedish Prescribed Drug Register was used to identify antibiotic treatment. Nationwide primary healthcare data and the National Patient Register (Out-patient data) were used to identify pyelonephritis. Model 1: Univariate. Model 2: Adjusted for individual sociodemographic factors. Model 3: Fully adjusted for all covariates.

Table S4. Sensitivity analysis on the association between antibiotic treatment (redeemed within two days) for acute uncomplicated cystitis and acute outpatient pyelonephritis within 7 days and 30 days from the cystitis event (2006-2018)

	**Total population**		**With acute pyelonephritis within 7 days**			**With acute pyelonephritis within 30 days**		
	No.	Proportion %	No.	Proportion %	Case rate %	No.	Proportion %	Case rate %
**No antibiotic treatment**	413 115	55.2	1597	60.1	0.39	2457	63.5	0.59
**Any antibiotics**[a]	335 589	44.8	1059	39.9	0.32	1412	36.5	0.42
Penicillins with extended spectrum (J01CA)	197 111	26.3	264	9.9	0.13	484	12.5	0.25
Nitrofuran derivatives (J01XE)	83 307	11.1	111	4.2	0.13	173	4.5	0.21
Trimethoprim and derivatives (J01EA)	34 554	4.6	38	1.4	0.11	49	1.3	0.14
Fluoroquinolones (J01MA)	15 781	2.1	591	22.3	3.75	640	16.5	4.06
Cephalosporins (J01DB-E,I)	3843	0.5	25	0.9	0.65	31	0.8	0.81
Others[b]	993	0.1	30	1.1	3.02	35	0.9	3.52
All	748 704	100.0	2656	100.0	0.35	3869	100.0	0.52

	**With acute pyelonephritis within 7 days**				**With acute pyelonephritis within 30 days**			
	**OR**[a]	**95% CI**		**P-value**	**OR**[a]	**95% CI**		**P-value**
**All antibiotic groups (ref. No treatment)**	0.82	0.76	0.89	<.0001	0.71	0.66	0.76	<.0001
**Specific antibiotic groups (ref. No treatment)**								
Penicillins with extended spectrum (J01CA)	0.35	0.30	0.39	<.0001	0.41	0.37	0.45	<.0001
Nitrofuran derivatives (J01XE)	0.34	0.28	0.42	<.0001	0.35	0.30	0.40	<.0001
Trimethoprim and derivatives (J01EA)	0.29	0.21	0.40	<.0001	0.24	0.18	0.32	<.0001
Fluoroquinolones (J01MA)	10.32	9.37	11.37	<.0001	7.18	6.57	7.85	<.0001
Cephalosporins (J01DB-E,I)	1.74	1.17	2.59	0.006	1.39	0.97	1.98	0.0704
Others[b]	8.22	5.70	11.87	<.0001	6.18	4.40	8.68	<.0001

CI: Confidence interval. OR: Odds ratio. Nationwide primary healthcare data was used to identify the study population of women with uncomplicated cystitis. The Swedish Prescribed Drug Register was used to identify antibiotic treatment within two days from the cystitis event but before the pyelonephritis event. Nationwide primary healthcare data and the National Patient Register (Out-patient data) were used to identify pyelonephritis. a: Fully adjusted for history of cervical cancer and parity, and individual sociodemographic factors. b: Other antibiotics included J01MB, J01EB, J01EC, J01ED, J01EE, and J01CR.

Table S5. Sensitivity analysis on the association between antibiotic treatment (redeemed within two days) for acute uncomplicated cystitis and acute outpatient pyelonephritis within 7 days and 30 days from the cystitis event, adjusted for sociodemographic factors, cervical cancer, and parity (2006-2018)

	**Pyelonephritis within 7 days**				**Pyelonephritis within 30 days**			
Covariates	OR[a]	95% CI		P-value	OR[a]	95% CI		P-value
**Antibiotic (ref. Non)**	0.82	0.76	0.89	<.0001	0.71	0.66	0.76	<.0001
**Age (ref. age 18-24 years)**								
25-34	0.91	0.80	1.03	0.1437	0.91	0.82	1.01	0.0642
34-44	0.97	0.85	1.12	0.7085	0.94	0.84	1.06	0.3248
45-65	0.89	0.78	1.01	0.0597	0.92	0.83	1.02	0.0968
**Educational level (ref. > 12 years)**	1.00	0.93	1.09	0.9338	1.05	0.98	1.13	0.1415
**Family income (ref. High)**								
Low	0.84	0.74	0.95	0.0040	0.89	0.81	0.98	0.0208
Middle	0.95	0.86	1.04	0.2676	0.96	0.89	1.04	0.3487
**Region of residence (ref. Large cities)**								
Southern Sweden	0.94	0.86	1.03	0.1830	0.96	0.89	1.03	0.2537
Northern Sweden	1.27	1.13	1.43	<.0001	1.15	1.03	1.27	0.0097
**Country of origin (ref. Born in Sweden)**								
Eastern Europe	0.85	0.70	1.04	0.1140	1.02	0.88	1.19	0.8010
Western countries	0.89	0.72	1.09	0.2518	0.96	0.81	1.13	0.6154
Middle East/North Africa	0.73	0.60	0.89	0.0023	0.78	0.66	0.92	0.0024
Africa (excluding North Africa)	0.88	0.63	1.22	0.4349	0.89	0.67	1.17	0.3924
Asia (excluding Middle East) and Oceania	1.09	0.86	1.37	0.4839	1.09	0.90	1.32	0.3968
Latin America and the Caribbean	0.83	0.58	1.18	0.2931	1.09	0.84	1.42	0.5060
**Cervical cancer (ref. Non)**	0.97	0.88	1.06	0.4739	0.97	0.90	1.05	0.5000
**Parity (ref. Non)**	1.06	0.85	1.32	0.6068	1.14	0.95	1.36	0.1645

CI: Confidence interval. OR: Odds ratio. Nationwide primary healthcare data was used to identify the study population of women with uncomplicated cystitis. The Swedish Prescribed Drug Register was used to identify antibiotic treatment. a: Fully adjusted for history of cervical cancer and parity, and individual sociodemographic factors.

Table S6 The study population of women with acute uncomplicated cystitis and hospitalization due to acute pyelonephritis (outcome) diagnosed within 30 days and 90 days from the cystitis event (2006-2018)

	Total population		With acute pyelonephritis within:					
			30 days			90 days		
	No.	Proportion %	No.	Proportion %	Case rate %	No.	Proportion %	Case rate %
Including the 1969 women with pyelonephritis prior to antibiotic treatment in the no antibiotic treatment group								
**No antibiotic treatment**	407 772	54.2	2355	91.5	0.58	2495	90.0	0.61
**Any antibiotic**[a]	344 517	46.8	219	8.5	0.06	278	10.0	0.08
**All**	752 289	100.0	2574	100.0	0.34	2773	100.0	0.37
Excluding the 1969 women with pyelonephritis prior to antibiotic treatment								
**No antibiotic treatment**	405 803	54.1	386	63.8	0.10	526	65.4	0.13
**Any antibiotic**[a]	344 517	45.9	219	36.2	0.06	278	34.6	0.08
Penicillins with extended spectrum (J01CA)	201 351	26.8	91	15.0	0.05	121	15.0	0.06
Nitrofuran derivatives (J01XE)	85 285	11.4	25	4.1	0.03	39	4.9	0.05
Trimethoprim and derivatives (J01EA)	35 369	4.7	15	2.5	0.04	16	2.0	0.05
Fluoroquinolones (J01MA)	17 275	2.3	81	13.4	0.47	94	11.7	0.54
Cephalosporins (J01DB-E,I)	4149	0.6	7	1.2	0.17	8	1.0	0.19
Others[b]	1088	0.1	0	0.0	0.00	0	0.0	0.00
**Age groups (years)**								
18-24	156 992	20.9	167	27.6	0.11	230	28.6	0.15
25-34	157 626	21.0	142	23.5	0.09	190	23.6	0.12
34-44	141 032	18.8	112	18.5	0.08	141	17.5	0.10
45-65	294 670	39.3	184	30.4	0.06	243	30.2	0.08
**Educational level**								
<12	467 080	62.3	388	64.1	0.08	518	64.4	0.11
> 12	283 240	37.7	217	35.9	0.08	286	35.6	0.10
**Family income**								
Low	179 621	23.9	135	22.3	0.08	186	23.1	0.10
Middle	380 486	50.7	331	54.7	0.09	427	53.1	0.11
High	190 213	25.4	139	23.0	0.07	191	23.8	0.10
**Region of residence**								
Large cities	482 822	64.3	405	66.9	0.08	530	65.9	0.11
Southern Sweden	194 500	25.9	129	21.3	0.07	176	21.9	0.09
Northern Sweden	72 998	9.7	71	11.7	0.10	98	12.2	0.13
**Country of origin**								
Sweden (born in)	596 865	79.5	493	81.5	0.08	654	81.3	0.11
Eastern Europe	36 332	4.8	31	5.1	0.09	45	5.6	0.12
Western countries	31 423	4.2	17	2.8	0.05	20	2.5	0.06
Middle East/North Africa	42 886	5.7	27	4.5	0.06	34	4.2	0.08
Africa (excluding North Africa)	11 868	1.6	11	1.8	0.09	13	1.6	0.11
Asia (excluding Middle East) and Oceania	20 357	2.7	16	2.6	0.08	26	3.2	0.13
Latin America and the Caribbean	10 589	1.4	10	1.7	0.09	12	1.5	0.11
**Parity**								
No child	319 165	42.5	277	45.8	0.09	388	48.3	0.12
With child	431 155	57.5	328	54.2	0.08	416	51.7	0.10
**Cervical cancer**								
Non	729 289	97.2	578	95.5	0.08	769	95.6	0.11
Yes	21 031	2.8	27	4.5	0.13	35	4.4	0.17
**All**	**750 320**	**100.0**	**605**	**100.0**	**0.08**	**804**	**100.0**	**0.11**

a: within five days from the cystitis event and before the pyelonephritis event. * 1969 women were excluded due to diagnosis of outcomes before antibiotic treatment. b: Others included ATC-codes J01MB, J01EB, J01EC, J01ED, J01EE, and J01CR. Nationwide primary healthcare data was used to identify the study population of women with uncomplicated cystitis. The Swedish Prescribed Drug Register was used to identify antibiotic treatment groups. The National Patient Register (In-patient data) were used to identify hospitalization due to pyelonephritis.

Table S7. The association between antibiotic treatment (redeemed within five days) for acute uncomplicated cystitis and hospitalization due to acute pyelonephritis within 30 days from the cystitis event, adjusted for sociodemographic factors, cervical cancer, and parity (2006-2018)

	Model 1				Model 2				Model 3			
Covariates	OR	95% CI		P-value	OR	95% CI		P-value	OR	95% CI		P-value
**Antibiotic (ref. Non)**	0.67	0.57	0.79	<.0001	0.65	0.55	0.77	<.0001	0.65	0.55	0.77	<.0001
**Age (ref. age 18-24 years)**												
25-34					0.84	0.67	1.06	0.1519	0.81	0.63	1.03	0.0893
34-44					0.74	0.58	0.94	0.0152	0.69	0.52	0.92	0.0105
45-65					0.57	0.46	0.71	<.0001	0.54	0.42	0.70	<.0001
**Educational level (ref. > 12 years)**					1.06	0.89	1.26	0.5239	1.05	0.89	1.25	0.5661
**Family income (ref. High)**												
Low					0.90	0.70	1.16	0.4080	0.89	0.69	1.15	0.3604
Middle					1.09	0.89	1.34	0.3867	1.09	0.89	1.33	0.4254
**Region of residence (ref. Large cities)**												
Southern Sweden					0.76	0.62	0.93	0.0077	0.76	0.62	0.93	0.0075
Northern Sweden					1.11	0.86	1.44	0.4132	1.11	0.86	1.43	0.4194
**Country of origin (ref. Born in Sweden)**												
Eastern Europe					1.10	0.77	1.60	0.5966	1.12	0.78	1.62	0.5376
Western countries					0.75	0.46	1.21	0.2362	0.76	0.47	1.23	0.2617
Middle East/North Africa					0.80	0.54	1.20	0.2818	0.82	0.55	1.23	0.3386
Africa (excluding North Africa)					1.15	0.63	2.10	0.6607	1.18	0.64	2.16	0.5963
Asia (excluding Middle East) and Oceania					1.00	0.61	1.65	0.9983	1.02	0.61	1.68	0.9545
Latin America and the Caribbean					1.20	0.64	2.24	0.5779	1.21	0.65	2.28	0.5477
**Cervical cancer (ref. Non)**									1.10	0.90	1.34	0.3422
**Parity (ref. Non)**									1.42	0.96	2.09	0.0800

CI: Confidence interval. OR: Odds ratio. Nationwide primary healthcare data was used to identify the study population of women with uncomplicated cystitis. The Swedish Prescribed Drug Register was used to identify antibiotic treatment. The National Patient Register (In-patient data) was used to identify hospitalization due to pyelonephritis. Model 1: Univariate. Model 2: Adjusted for individual sociodemographic factors. Model 3: Fully adjusted for all covariates

Table S8. The association between antibiotic treatment (redeemed within five days) for acute uncomplicated cystitis and hospitalization due to acute pyelonephritis within 90 days from the cystitis event, adjusted for sociodemographic factors, cervical cancer, and parity (2006-2018)

	Model 1				Model 2				Model 3			
Covariates	OR	95% CI		P-value	OR	95% CI		P-value	OR	95% CI		P-value
**Antibiotic (ref. Non)**	0.62	0.54	0.72	<.0001	0.60	0.52	0.70	<.0001	0.61	0.52	0.70	<.0001
**Age (ref. age 18-24 years)**												
25-34					0.81	0.66	0.99	0.0395	0.81	0.66	1.00	0.0505
34-44					0.67	0.54	0.83	0.0002	0.67	0.53	0.86	0.0017
45-65					0.54	0.45	0.65	<.0001	0.55	0.44	0.69	<.0001
**Educational level (ref. > 12 years)**					1.06	0.91	1.24	0.4337	1.06	0.91	1.23	0.4448
**Family income (ref. High)**												
Low					0.89	0.71	1.10	0.2823	0.89	0.71	1.11	0.2981
Middle					1.02	0.85	1.21	0.8710	1.02	0.85	1.21	0.8619
**Region of residence (ref. Large cities)**												
Southern Sweden					0.79	0.67	0.94	0.0082	0.79	0.67	0.94	0.0083
Northern Sweden					1.17	0.94	1.46	0.1516	1.17	0.94	1.46	0.1544
**Country of origin (ref. Born in Sweden)**												
Eastern Europe					1.23	0.90	1.67	0.1923	1.23	0.90	1.67	0.1901
Western countries					0.67	0.43	1.05	0.0786	0.67	0.43	1.05	0.0809
Middle East/North Africa					0.76	0.54	1.09	0.1373	0.77	0.54	1.10	0.1513
Africa (excluding North Africa)					1.02	0.59	1.78	0.9399	1.03	0.59	1.80	0.9123
Asia (excluding Middle East) and Oceania					1.25	0.84	1.85	0.2762	1.25	0.84	1.86	0.2686
Latin America and the Caribbean					1.10	0.62	1.96	0.7361	1.11	0.62	1.96	0.7288
**Cervical cancer (ref. Non)**									0.99	0.84	1.17	0.9031
**Parity (ref. Non)**									1.37	0.97	1.93	0.0707

CI: Confidence interval. OR: Odds ratio. Nationwide primary healthcare data was used to identify the study population of women with uncomplicated cystitis. The Swedish Prescribed Drug Register was used to identify antibiotic treatment. The National Patient Register (In-patient data) was used to identify hospitalization due to pyelonephritis. Model 1: Univariate. Model 2: Adjusted for individual sociodemographic factors. Model 3: Fully adjusted for all covariates.

Figure S1. Flowchart of the eligibility criteria and sampling of the study population of women with a diagnose (first occurrence) of acute uncomplicated cystitis in nationwide primary healthcare data (1st January 2006 to 2nd October 2018) and follow-up for pyelonephritis (31st December 2018)

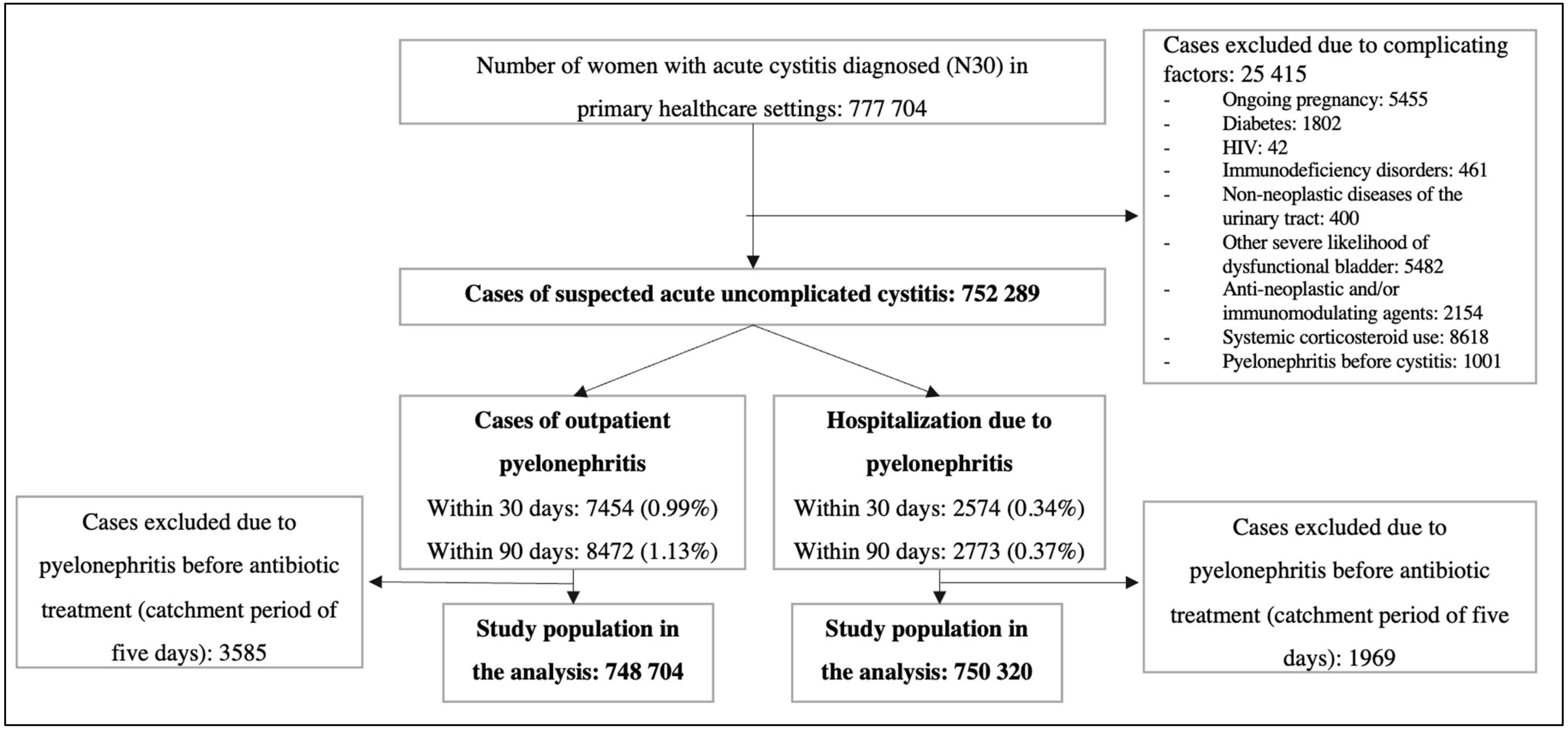

Figure S2. The 30-day risk of acute outpatient pyelonephritis after acute uncomplicated cystitis treated with antibiotics or not (redeemed within two days)
(survival probability 1.00 = no cases of pyelonephritis)

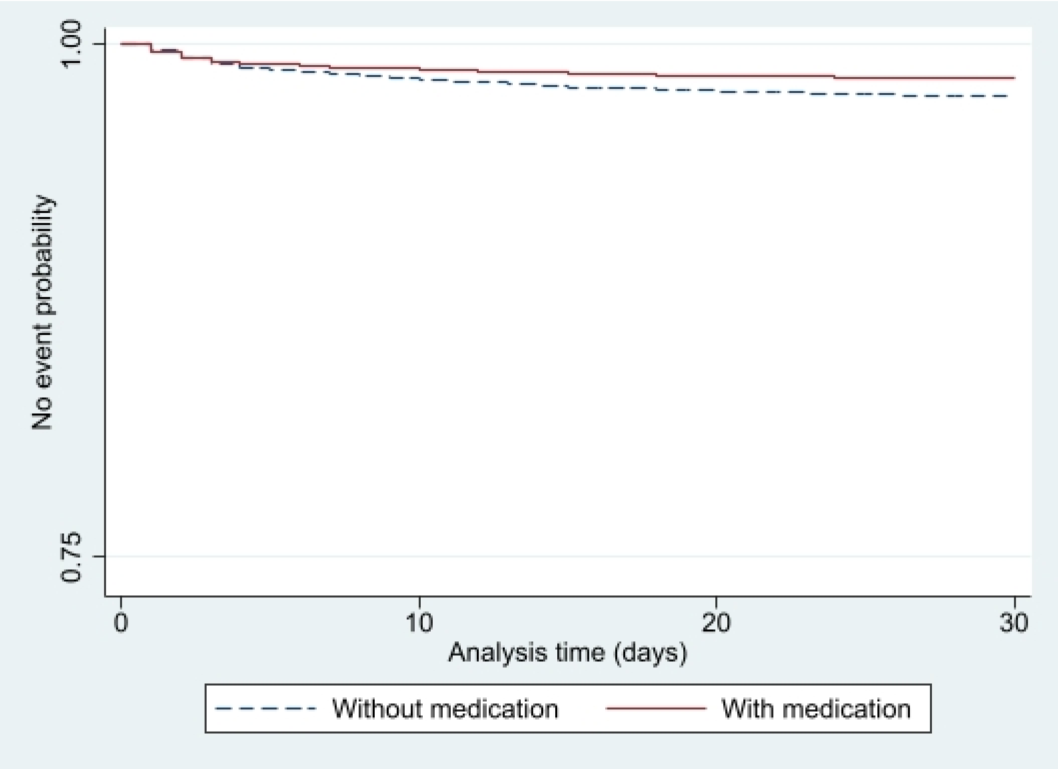

Figure S3. The 30-day risk of acute outpatient pyelonephritis after acute uncomplicated cystitis treated with antibiotics or not (redeemed within five days)
(survival probability 1.00 = no cases of pyelonephritis)

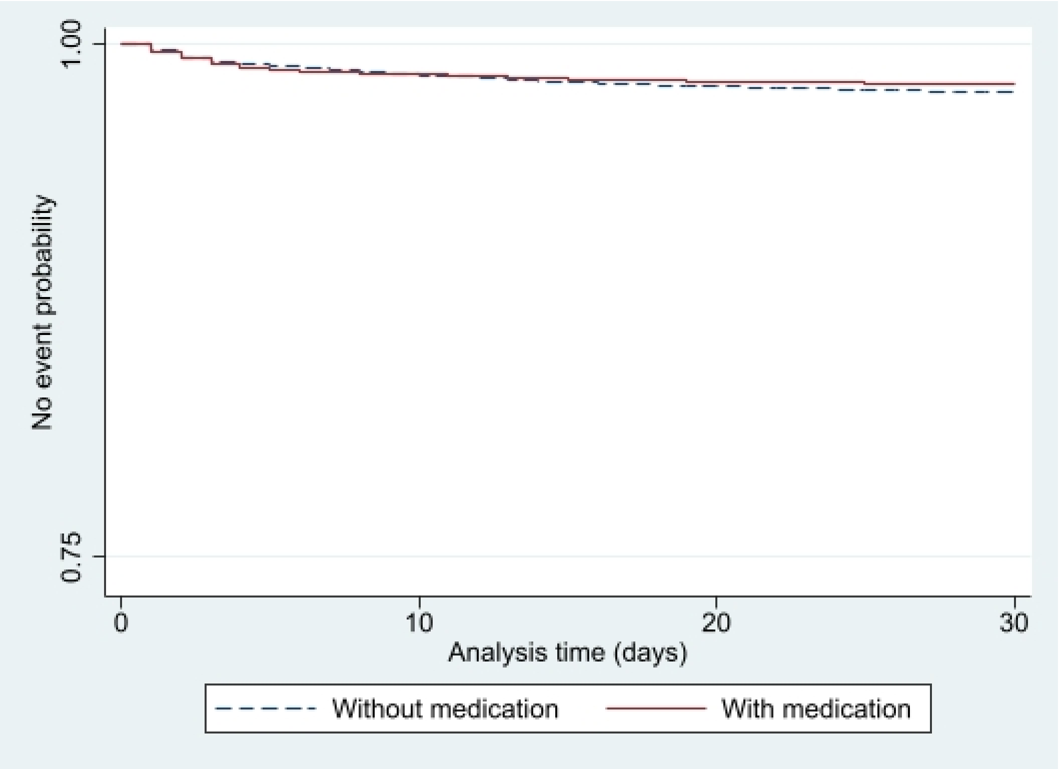

Figure S4. The 30-day risk of hospitalization due to acute pyelonephritis after acute uncomplicated cystitis treated with antibiotics or not (redeemed within five days)
(survival probability 1.00 = no cases of pyelonephritis)

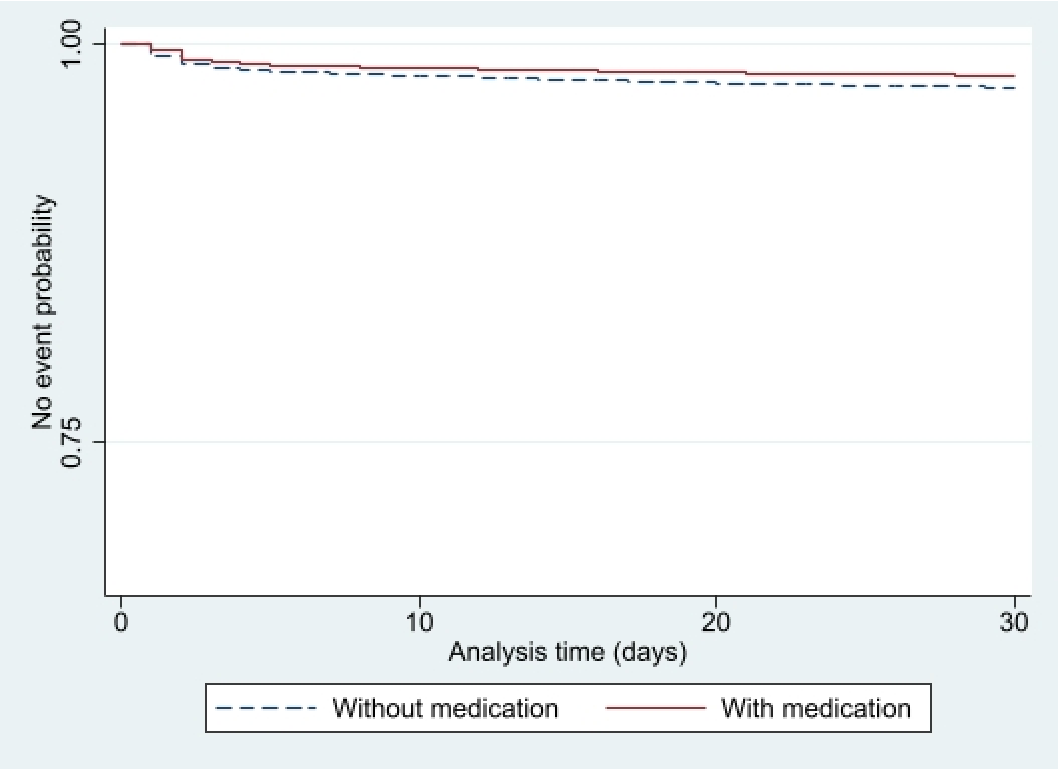

Supplement: Supplementary file 1 [file antibiotics-11-01695-s001.zip › antibiotics-2017285-supplementary.pdf]
